# Prostatic Abscess after Stapled Hemorrhoidopexy Caused by ESBL Extended Spectrum Beta Lactamase Producing* Klebsiella pneumoniae*: An Additional Challenge to Postoperative Sepsis

**DOI:** 10.1155/2017/4154016

**Published:** 2017-07-17

**Authors:** Asem Saleh, Abdelnasir Kibeida, Elsaid Amin, Abdalla Khalil, Rafat Abu Shakra, Mohamed Elwakil

**Affiliations:** ^1^International Medical Centre (IMC) Hospital, Internal Medicine Department, Jeddah, Saudi Arabia; ^2^IMC Hospital, General Surgery Department, Jeddah, Saudi Arabia; ^3^IMC Hospital, Radiology Department, Jeddah, Saudi Arabia; ^4^IMC Hospital, Clinical Pathology Department, Saudi Arabia; ^5^IMC Hospital, Emergency Medicine Department, Jeddah, Saudi Arabia

## Abstract

Postoperative septic complications of hemorrhoids surgical interventions are rare, but very serious with high mortality rate. Early diagnosis and prompt therapy are essential to save patient's life. There are a good number of articles and case reports about these septic complications. We are presenting a case report of a prostatic abscess caused by extended spectrum beta lactamase (ESBL) producing* Klebsiella pneumoniae* after hemorrhoidopexy. Our patient was a healthy middle aged Saudi male who has no significant medical history apart from morbid obesity and recurrent urinary tract infections. ESBL producing* K. pneumoniae* could be detected only after aspiration of the prostatic abscess, but proper antibiotic was introduced intravenously on admission before culture of aspirate of the abscess was available. Antibiotic was continued for 30 days and abscess resolved completely. In our electronic search, we could not find any case report of prostatic abscess after stapled hemorrhoidopexy caused by ESBL producing organism. This is an additional challenge for treating physicians as these organisms are sensitive only to one group of antibiotics (carbapenem group).

## 1. Introduction

Septic complications after treatment of hemorrhoids are extremely rare, but these can be devastating and have resulted in a number of deaths [1].

Retroperitoneal abscess and sepsis have been reported after SH stapled hemorrhoidopexy [2, 3].

Perineal necrosis, abscess, and septic shock have been also reported after SH [4, 5].

Sepsis following hemorrhoids treatment has been reported as early as 12 hours after therapy [2].

It can take up to 7 days to manifest clinically and a rare case of severe perineal sepsis has been reported after 38 days following SH [4, 6].

Sepsis, septic shock, and liver abscess have been also reported with other modalities of treating hemorrhoids as sclerotherapy and rubber band ligation [7, 8].

A retrospective study showed that septic complications after hemorrhoidectomy represent only 0.1% of all operated on patients (2840 patients) [9].

Studies showed that SH has a significant higher rate of recurrence and postprocedural tenesmus [10].

## 2. Case Presentation

49-year-old Saudi male was operated on electively for second- and third-degree hemorrhoids after two ER visits with rectal blood spotting. His past medical and surgical history were unremarkable apart from morbid obesity and recurrent urinary tract infections that required intravenous therapy in other hospital.

Indications for surgery were recurrent bleeding with second- and third-degree hemorrhoids.

Surgery was done under spinal anesthesia and intravenous sedation.

No Foley's catheter was inserted. Patient was placed in lithotomy posture.

Examination under general anesthesia revealed second- and third-degree hemorrhoids.

Procedure started by placing the anal dilator and fixing it with 2/0 silk interrupted sutures.

Purse string anoscope was then used to place the purse string sutures on rectal submucosal level using Prolene 2/0, placing 5-6 bites at 4 cm distance from the anal verge.

Stapler device 33 mm Ethicon proximate (fixed anvil) stapler was then introduced and purse string snugly tied; the Prolene suture ends were then retrieved through the device housing using the suture threader.

Device was closed by marking and then fired.

An evenly excised mucosa was then retrieved, width of which is about 1.5 cm.

Hemostasis was well secured with interrupted vicryl 3/0 sutures.

No major complications were present during the procedure.

As the patient weight was 114 kgm, height 172 cm, and BMI 38.5., the only difficulty encountered during surgery was in placing the anal dilator and fixing it in a correct position.

The patient was stable during the procedure with minimal blood loss and was discharged to the postanesthesia recovery room.

Five days after discharge, he came back to ER with fever, dysuria, frequency, and urgency for two days. His temperature was 38.2°C and the rest of exam was unremarkable. His complete blood count and renal function tests were normal. Urinalysis showed leucocytes +2, WBC 40/HPF, and negative nitrates.

He was treated as acute cystitis with ciprofloxacin 500 mg twice daily orally.

He visited ER again after another two days with fever, rigors, abdominal pain, repeated vomiting, and generalized weakness. No rectal bleeding, pain, or discharge was present.

He denied any history of extramarital sexual relation or sexually transmitted disease before.

By examination, he looked sick and dehydrated. Temperature was 38.9°C, BP 110/70, pulse 120/min regular, and oxygen saturation 97% at room air.

His abdominal exam showed tender suprapubic area and perineum. There was no discharge or bleeding from anal orifice. Cardiac and respiratory exam were unremarkable.

His CBC complete blood count and renal function tests were normal. Urinalysis showed leucocytes +3, WBC > 100/HPF with negative nitrates.

Urine culture of previous ER visit was negative.

His CRP was raised 64 mg/l (0–5 mg/l) and PSA total was 6 ug/l (0–4.4 ug/l).

Both blood culture and urine cultures came to be negative. Both Chlamydia and gonococci antibody were negative.

Histopathology of the previous resected hemorrhoids specimen was reviewed.

**Figure 1:**
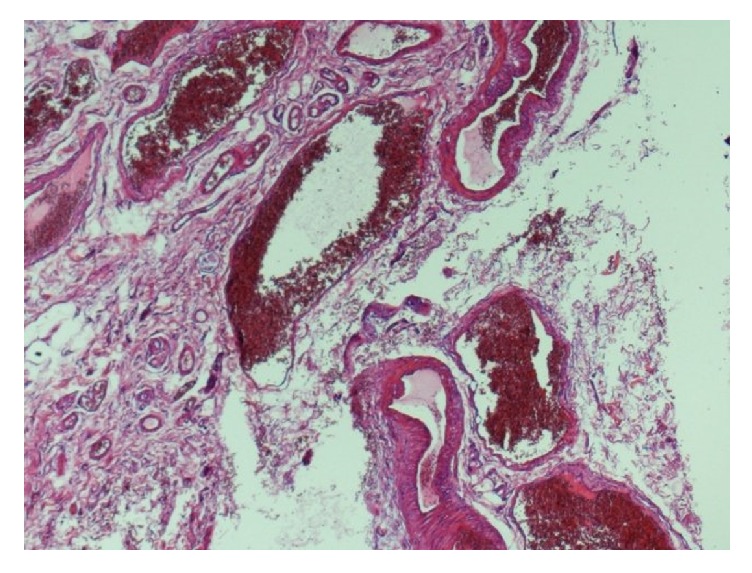
Photomicrograph shows hemorrhoids comprised of dilated, thick walled, congested submucosal blood vessels (hematoxylin and eosin stain, original magnification ×40).

**Figure 2:**
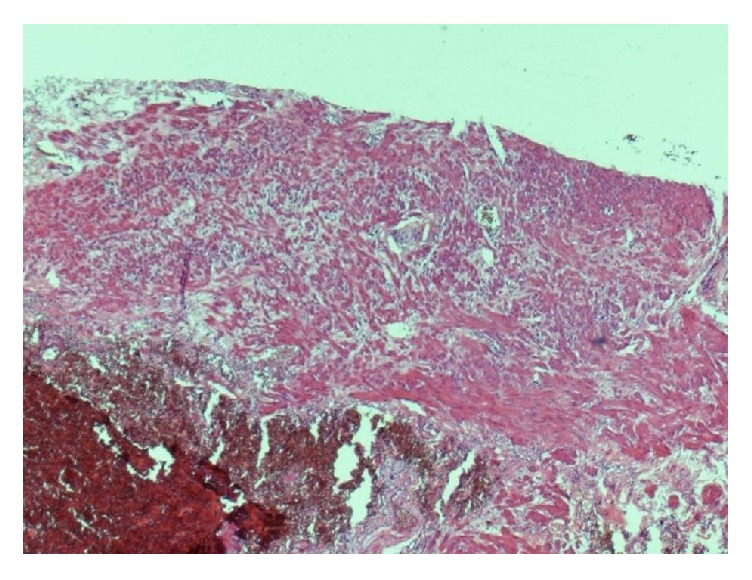
Photomicrograph shows portion of rectal muscularis propria identified at the deep aspect of the specimen (hematoxylin and eosin stain, original magnification ×40).

It showed hemorrhoids comprised of dilated, thick walled, congested submucosal blood vessels (Figure 1). A portion of rectal muscularis propria was identified at the deep aspect of the specimen (Figure 2).

Abdomen and pelvis ultrasound at ER were unremarkable apart from enlarged prostate 45 cc. Patient was started empirically on meropenem intravenously 1 gm every 8 hours and he continued to be febrile for another 2 days.

**Figure 3:**
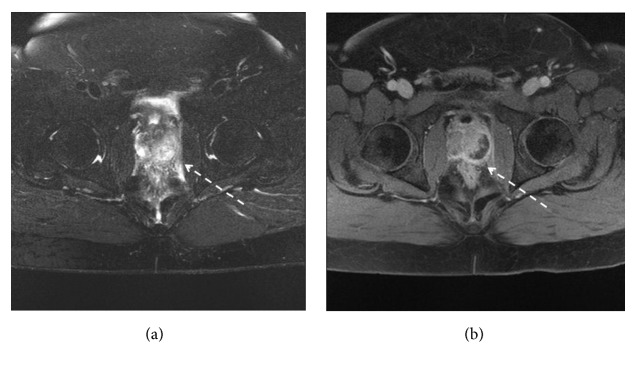
MRI of pelvis: axial FS T2WI (a), axial postcontrast T1WI (b). Before aspiration and antibiotic therapy showing fluid intensity lesion within posterolateral aspect of the gland measuring 24 × 12 × 14 mm with marginal enhancement (prostatic abscess).

On third day after admission, MRI of pelvis showed fluid intensity lesion within posterolateral aspect of the prostate gland measuring 24 × 12 × 14 mm with marginal enhancement (prostatic abscess) (Figure 3).

Infectious disease consultant and urologist suggested ultrasound guided transrectal aspiration of the abscess.

Purulent fluid of 5 ml was aspirated and sent for microbiology. It grew gram negative bacilli* Klebsiella pneumoniae* (morphology was done according to CDC algorithm).* Klebsiella pneumoniae* was resistant to cefuroxime, ceftazidime, ceftriaxone, and piperacillin/tazobactam and sensitive to ciprofloxacin, meropenem, imipenem, and gentamicin, that is, extended spectrum beta lactamase (ESBL) producing (culture sensitivity was done; MIC results obtained using automated Vitek 2 AST-GN69 and AST –XN06 cards).

Fever subsided next day after aspiration of abscess and patient felt well.

**Figure 4:**
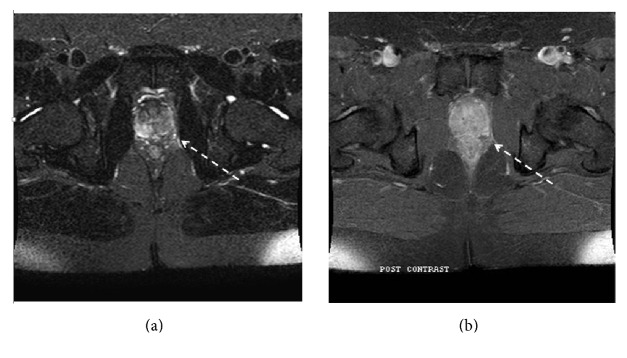
MRI of pelvis: axial FS T2WI (a), axial postcontrast T1WI (b). After aspiration and antibiotic therapy showing resolution of the previous intraprostatic collection.

Meropenem continued for total of 14 days and then changed to ciprofloxacin 500 mg bid and clindamycin 300 mg q8 hours orally for total of 30 days. He was followed at infectious diseases clinic and repeated CRP was 6 mg (0–5 mg/l) and MRI showed resolution of the previous intraprostatic collection with residual edema along left perianal area due to previous surgery (Figure 4).

## 3. Discussion

Sepsis caused by MDR multidrug resistant bacteria represents an additional challenge to postoperative septic complications of hemorrhoids treatment.

Detection of these MDR bacteria will take 72 hours till we have culture sensitivity of aspirated fluid or blood culture and this will be reflected on outcome of the septic patient.

Extended spectrum beta lactamase (ESBL) producing Enterobacteriaceae (e.g.,* Escherichia coli and Klebsiella pneumoniae*) is one of the challenging hospital acquired infections worldwide [11].

ESBL producing* Escherichia coli (E. coli) and Klebsiella pneumoniae *infections carry a higher mortality rate, higher risk of developing bacteremia, and failure of therapy compared to nonproducing ESBL isolates [12, 13].

Carbapenem group of antibiotics is the drug of choice for treating* ESBL E. coli and Klebsiella pneumoniae *infections.

Prostatic abscess after SH in our case report could be explained by the possibility that purse string might have been a bit too profound, on the anterior side, taking not only mucosa and submucosa, but also a bite of rectal smooth muscle (Figures 1 and 2).

In our electronic search, we could not find any reported prostatic abscess caused by ESBL* E. coli or Klebsiella pneumoniae *infection as postoperative septic complications for hemorrhoids surgical intervention.

Our patient presented with fever and symptoms of genitourinary tract infection five days after hemorrhoidopexy. His urinalysis was nitrates negative and WBCs were 40/HPF, but urine culture was negative.

Treating team considered possibility of ESBL with patient's past history of recurrent urinary tract infections and intravenous therapy at other hospital.

Patient was started on meropenem empirically. Both blood culture and urine culture on admission came to be negative while purulent fluid aspirated from prostatic abscess grew ESBL* K. pneumoniae*.

With aspiration of the prostatic abscess, causative organism could be detected and resolution of the abscess could be achieved after continuing the proper antibiotic therapy.

Our surgical site infection rate percentage at our hospital is 0.1–0.4% which comes with the acceptable international rate [14].

## Abbreviations

ESBL: Extended spectrum beta lactamase
MDR: Multidrug resistance
ER: Emergency room
HPF: High power field
SH: Stapled hemorrhoidopexy.
